# High Peritoneal Transport Status Was Not Associated with Mortality in Peritoneal Dialysis Patients with Diabetes

**DOI:** 10.1371/journal.pone.0110445

**Published:** 2014-10-16

**Authors:** Naya Huang, Jiehui Chen, Li Fan, Qian Zhou, Qingdong Xu, Ricong Xu, Liping Xiong, Xueqing Yu, Haiping Mao

**Affiliations:** 1 Department of Nephrology, The First Affiliated Hospital, Sun Yat-sen University, Key Laboratory of Nephrology, Ministry of Health of China, Guangdong Provincial Key Laboratory of Nephrology, Guangzhou, Guangdong, China; 2 Department of Nephrology, The Sixth People's Hospital of Shenzhen, Shenzhen, Guangdong, China; 3 Epidemiology Research Unit, Translational Medicine Research Center, The First Affiliated Hospital, Sun Yat-sen University, Guangzhou, Guangdong, China; University of Utah School of Medicine, United States of America

## Abstract

**Background:**

Continuous ambulatory peritoneal dialysis (CAPD) patients with diabetes are at increased risk of mortality and high peritoneal transporters appear to contribute to poor survival. However, little is known about the combined impacts of high peritoneal transporters and diabetes on mortality.

**Methods:**

This was a prospective observational cohort study. 776 incident CAPD patients were enrolled. Unadjusted and adjusted Cox proportional regression models were used to evaluate the association and interaction of peritoneal transport and diabetic status with mortality

**Results:**

In the entire cohort, high peritoneal transport status was associated with an increased risk of all-cause mortality in unadjusted model [hazard ratio (HR) 2.35, 95% confidence interval (CI) 1.30 to 4.25, P = 0.01], but this association was not significant in multivariable model. There was an interaction between peritoneal membrane transport status and diabetes (*P* = 0.028). Subgroup analyses showed that compared to low and low average transporters, high transporters was associated with a higher risk of all-cause mortality (adjusted HR 1.78, 95% CI 1.07 to 4.70, *P* = 0.04) in CAPD patients without diabetes, but not in those with diabetes (adjusted HR 0.79, 95%CI 0.33 to 1.89, *P* = 0.59). Results were similar when transport status was assessed as a continuous variable.

**Conclusions:**

The association between high peritoneal transport and all-cause mortality was likely to vary with diabetes status. High peritoneal transport was associated with an elevated risk of death among CAPD patients without diabetes, but not in those with diabetes.

## Introduction

Peritoneal transport characteristics, as assessed by the dialysate to plasma ratio of creatinine (D/P Cr) from a peritoneal equilibration test (PET), is traditionally used to evaluate peritoneal permeability in peritoneal dialysis (PD) setting [1]. However, peritoneal transport status has been demonstrated to have a paradoxical association with mortality in PD patients. Many previous studies [2]–[6], such as the CANUSA study and the study from Australian and New Zealand, indicated that high peritoneal transporters had adverse impacts on both mortality and technique failure in PD patients. However, these findings have not been confirmed by other studies [7]–[10]. In the ADEMEX trial, a prospective study, showed that high transport had no impact on patient survival in PD patients. Meanwhile, a Korean study has shown that peritoneal protein clearance, which is positively correlated with D/P Cr, is not associated with increased all-cause mortality in multivariate Cox analysis in PD patients [11]. The discrepancy between the conflicting results is not clearly demonstrated, but may due to difference in the primary cause of end stage of renal disease (ESRD), a mixture of incident and prevalent patients, racial origin, the methodology of transport test, and comorbid illness burden.

Diabetes mellitus (DM) is increasing to be the second cause of ESRD in China. Compared with PD patients without diabetes, those with diabetes generally have poorer patient survival. A number of studies have suggested that diabetes *per se* is associated with a high risk of mortality [12]–[14]. Meanwhile, high peritoneal transporters have been shown to be more prevalent in PD patients with diabetes [2], [15]. However, it is unknown whether high peritoneal transporters contribute additional mortality risk in PD patients with diabetes.

In this study, we evaluated the association of baseline peritoneal transporters and diabetic status on all-cause mortality in CAPD patients and explored whether this association varied by diabetes mellitus.

## Materials and Methods

### Study Design and Population

This study protocol was approved by the Ethics Committee of The First Affiliated Hospital of Sun Yat-sen University. Written informed consent form was obtained from all participants.

It was a prospective observational cohort study. A total of 776 incident patients who commenced CAPD in our hospital between January 2007 and December 2011 were studied. Patients older than 18 years old undergoing CAPD treatment for more than 3 months were included. Patient who had a history of kidney transplantation, hemodialysis for more than 3 months, and malignant disease was excluded.

A standard PET and total weekly creatinine clearance (peritoneal and residual renal clearance) and urea Kt/V were performed as previous described [14]. Briefly, a standard 4-hour dwell period was used, with a 2.27% glucose concentration 2 L volume exchange. The results were calculated by using PD Adequest software (Baxter Healthcare Corporation, Chicago, IL, USA). The first PET tests were performed between the first and third month post-PD initiation in patients without significant fluid overload or acute peritonitis and used for analyses. Peritoneal transport status was categorized as low (L), low average (LA), high average (HA), and high (H), according to D/P Cr 4h values defined by Twardowski (L<0.50, LA 0.5–0.64, HA 0.65–0.80, H≥0.81)[16]. We used peritoneal transport status as a categorical variable (PET classes) in our primary analyses, and a continuous variable (D/P Cr 4h) in sensitivity analyses. L transporters (n = 25, 3.2%) were combined into LA transporters (L&LA) for analyses due to relatively few patients.

To investigate whether the relationship between peritoneal transport status and mortality varied in patients with diabetes, subjects were divided into groups with diabetes mellitus (DM) and without diabetes mellitus (NDM) according to the presence or absence of diabetes. Diabetic status was defined if diabetes mellitus was registered as the primary cause of ESRD (included type 1 or 2) or patients with diabetes as comorbidity.

### Demographic and Clinical Data

Baseline demographic data included the age, gender, primary cause of ESRD, the presence of cardiovascular disease (CVD) and diabetic status. Cardiovascular disease was defined as myocardial infarction, angina, arrhythmias, valvular heart disease, congestive heart failure, ischemic stroke, transient ischemic attack, peripheral arterial disease [17], [18] . Residual renal function (RRF) was defined as the average of 24-h urinary urea and creatinine clearance, and the ultrafiltration volume (UF) was calculated as the difference of infused and drained dialysate volume during PET. The clinic and biochemical data were collected at the first 3 months after PD initiation.

### Outcomes

The primary outcome of this study was all-cause mortality. Survival time was defined as the time from enrollment to death or administrative censoring (transfer to hemodialysis, kidney transplantation, transfer to other dialysis centers, loss of follow-up, or end of the study period) at December 31, 2012.

### Statistical analysis

Results of continuous variables were expressed as mean ± standard deviation (SD) for normal distribution, median (interquartile range, IQR) for skewed distribution. For categorical variables, the results were expressed as frequencies and percentages. Comparison of continuous variables and categorical data between two groups in the study were tested by t-test and Chi-Square test.

Survival curves were generated by Kaplan-Meier method. Because patients with diabetes were much older than the non-diabetic ones, we plotted the survival curves both in the entire cohort and in the cohort with age and gender matched patients to demonstrate the patient survival in diabetic and non-diabtetic CAPD patient. Unadjusted and adjusted Cox proportional regression models were used to evaluate the association of peritoneal transport status and all-cause mortality. Variables with *P*-value <0.1 in univariate models and RRF (Table S1) were selected for multivariate Cox regression models.

Given that CAPD patients with diabetes had more proportion of H transporters and poorer survival than those without diabetes, we evaluated *a prior* interaction between peritoneal transport status and diabetes using multivariable Cox regression analyses. Then, we perform subgroup analyses to evaluate the relationship of peritoneal transport status and all-cause mortality in individuals with or without diabetes. All statistical analyses were performed using the SPSS software, version 16.0 (SPSS Inc., Chicago, IL, USA). A *P*-value<0.05 was considered statistically significant.

## Results

### Baseline characteristics of the participants

Table 1 showed the baseline demographic and clinical characteristics of the entire cohort and stratified by diabetic status. Overall, the mean age was 46.85±15.12 years, 58.1% were male, and 22.6% had diabetes. The mean D/P Cr 4h was 0.71±0.12 and there were 237 (30.5%) patients with L&LA transport, 373 (48.1%) with HA, and 166 (21.4%) with H transport. The baseline mean D/P Cr 4h was significant higher in patients with diabetes than those without diabetes (0.75±0.12 *vs.* 0.70±0.11; *P*<0.001); and CAPD patients with diabetes had higher proportion of H transporters. In addition, participants with diabetes were more likely to be older, had a greater percentage of CVD, and higher levels of body mass index (BMI), hemoglobin A1c, triglyceride and RRF, and lower diastolic pressure, hemoglobin, serum albumin, serum prealbumin, normalized protein catabolic rate (nPCR), serum uric acid, phosphate and intact parathyroid hormone (iPTH). However, there were no significant differences between DM and NDM group with respect to the total weekly creatinine clearance, total weekly Kt/V urea, and ultrafiltration volume.

**Table 1:** Baseline demographic and clinical characteristics of the study population.

Variables	Total (n = 776)	NDM (n = 600)	DM (n = 176)	*P*
PET classes				**0.002**
L&LA	237 (30.5%)	193 (32.2%)	44 (25.0%)	
HA	373 (48.1%)	296 (49.3%)	77 (43.8%)	
H	166 (21.4%)	111 (18.5%)	55 (31.2%)	
D/P Cr 4h	0.71±0.12	0.70±0.11	0.75±0.12	**<0.001**
Age (year)	46.85±15.12	43.54±14.70	58.17±10.25	**<0.001**
Male gender	451 (58.1%)	350 (58.3%)	101 (57.1%)	0.77
Dialysis vintage	26.3(16.5, 40.2)	26.0(16.4, 39.3)	27.0(17.3, 42.4)	0.21
History of cardiovascular disease	361 (46.5%)	253 (41.9%)	108 (61.4%)	**<0.001**
BMI (kg/m^2^)	21.05±3.93	20.65±3.79	22.42±4.08	**<0.001**
SBP (mmHg)	137.38±17.07	136.71±17.20	139.56±16.47	0.06
DBP (mmHg)	82.84±12.28	84.51±12.40	77.14±9.96	**<0.001**
Hemoglobin (g/L)	109.9±21.5	111.0±21.7	106.3±20.0	**0.01**
Serum albumin (g/L)	37.6±5.9	38.3±5.6	35.3±6.1	**<0.001**
Serum prealbumin(mg/L)	347.48±102.92	365.32±99.34	289.10±92.58	**<0.001**
nPCR (g/kg/d)	0.94±0.23	0.96±0.23	0.86±0.22	**<0.001**
Uric acid (µmol/L)	406.2±91.0	412.2± 86.8	381.3±98.7	**<0.001**
Calcium (mmol/L)	2.30±0.36	2.30±0.29	2.29 ±0.56	0.93
Phosphate(mmol/L)	1.46± 0.48	1.48±0.51	1.37 ±0.34	**0.002**
iPTH (pg/mL)	241 (103, 416)	247 (108, 433)	210 (82, 371.5)	**0.02**
Glucose (mmol/L)	6.34±1.13	5.85±1.07	8.02±1.32	0.26
Haemoglobin A1c(%)	6.29 ±1.26	6.58±1.23	6.08±1.27	**0.008**
Cholesterol (mmol/L)	5.29±2.97	5.12±2.05	5.89±4.87	**0.05**
Triglyceride(mmol/L)	1.71±1.17	1.58±0.90	2.12±1.73	**<0.001**
hs-CRP (mg/L)	1.74 (0.66, 6.47)	1.64 (0.66, 5.74)	2.23 (0.65, 8.58)	0.30
UF on PET (ml)	280 (160, 380)	300 (160, 380)	240 (120, 380)	0.20
WCCr(L/w/1.73 m^2^)	88.83±35.37	88.42±5.54	90.20±28.61	0.82
Weekly total Kt/V urea	2.40±0.64	2.38±0.67	2.46±0.57	0.16
RRF (ml/min/1.73 m^2^)	3.13 (1.86, 5.02)	3.02 (1.74, 4.56)	4.06 (2.39, 5.90)	**0.001**

Results are expressed as mean ± SD, median (interquartile range) or number (%).

Abbreviations: BMI, body mass index; SBP, systolic blood pressure; DBP, diastolic blood pressure; nPCR, normalized protein catabolic rate; iPTH, intact parathyroid hormone; hs-CRP, high-sensitivity C-reactive protein; PET, peritoneal equilibration test; UF, ultrafitration; WCCr, total weekly creatinine clearance; RRF, residual renal function; NDM, non-diabetes mellitus, DM, diabetes mellitus; H, high transport; HA, high average transport; LA, low average transport; L, low transport.

### Patient survival

**Figure 1:**
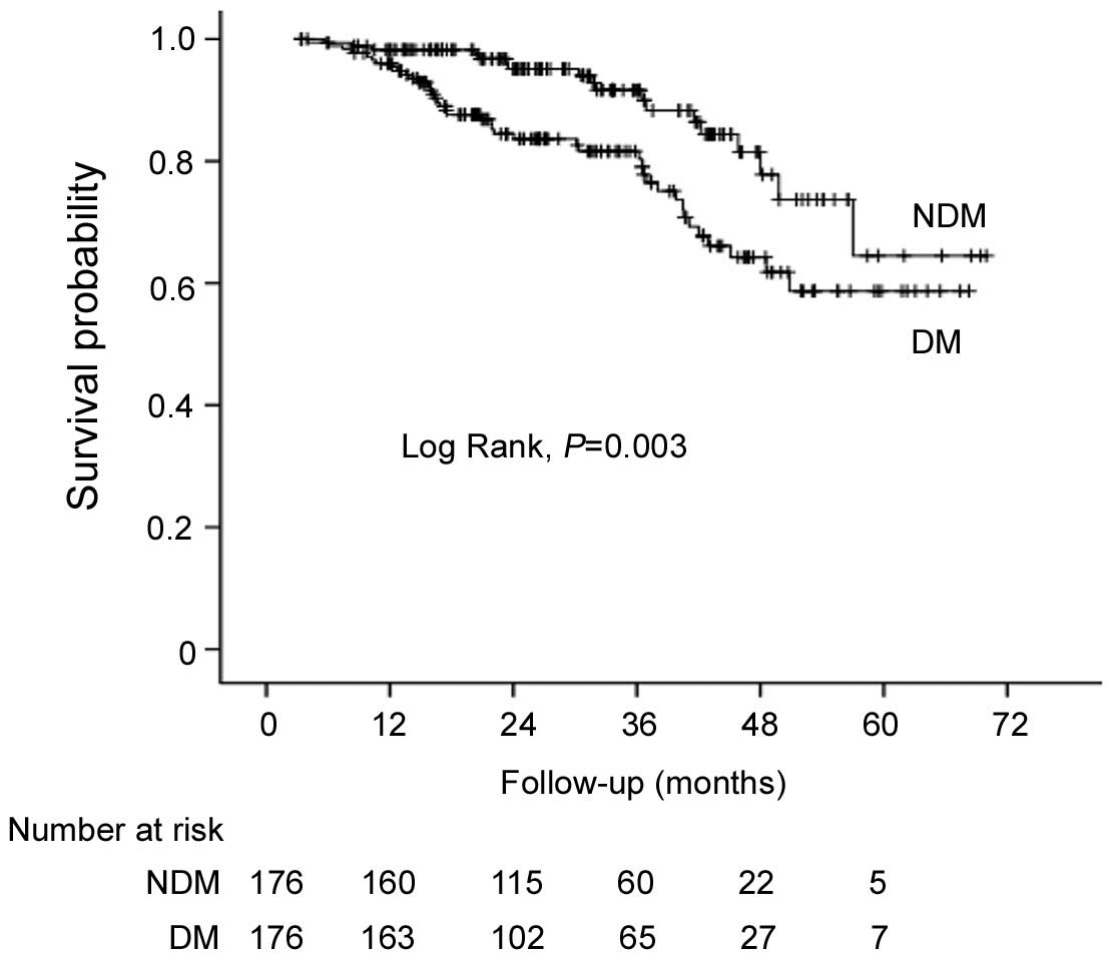
Kaplan-Meier survival curves for age and sex matched CAPD patients with and without diabetes.

During a median follow-up of 26.3 months (IQR: 16.5, 40.2), 76 patients (9.8%) died, in which 35 (5.8%) were in NDM and 41 (23.3%) in DM group. In the cohort with age- and gender-matched patients, Kaplan-Meier curve showed that diabetic CAPD patients had significant poorer patient survival compared to those without diabetes (Figure 1, *P* = 0.003). Similar results were shown in the entire cohort (data not shown). The cumulative survival rates at 1-, 3- and 5- year were 96%, 81%, and 56% in diabetic patients, and 99%, 94%, and 83% in non-diabetic patients, respectively.

### Interaction of peritoneal transport status and diabetes and their association with mortality

Table 2 demonstrated the association of transport status, diabetes and all-cause mortality in entire cohort. Compared to L&LA transporters, H transporters was associated with an increased risk of all-cause mortality in the unadjusted model [hazard ratio (HR) 2.35, 95% confidence interval (95% CI) 1.30 to 4.25; *P*-trend = 0.003]. However, this relationship was attenuated and not significant after adjustment for potential confounders (adjusted HR 1.31, 95% CI 0.68 to 2.54; *P*-trend = 0.13). Results were similar when transport status was evaluated as a continuous variable. Further, we assessed the interaction between peritoneal transporters and diabetes on patient survival. The data demonstrated that a significant interaction between transport status and diabetes when PET was evaluated as a categorical variable (*P*-interaction = 0.028), and a continuous variable (*P*-interaction  = 0.048).

**Table 2:** Interaction and association of transport status, diabetes and all-cause mortality in entire cohort.

Variable	Unadjusted model, HR (95% CI)	Adjusted model*, HR (95% CI)	Adjusted interaction *P*-value between transport status and diabetes*
Transport status as a categorical variable			**0.028**
PET Classes			
L&LA	Ref	Ref	
HA	1.07 (0.60, 1.92)	0.74 (0.40, 1.36)	
H	2.35 (1.30, 4.25)	1.31 (0.68, 2.54)	
* P*- trend	**0.003**	0.13	
Transport status as a continuous variable			**0.048**
D/P Cr 4h (per 0.1 higher)	1.42 (1.17, 1.72)	1.18 (0.95, 1.48)	

*Adjusted for the variables with *P*<0.1 in the univariate model and RRF, including age at initiation of peritoneal dialysis, gender, cardiovascular disease, hemoglobin, serum albumin, DBP, iPTH, total cholesterol, and triglyceride and RRF (Table S1). Transport status was included in the adjusted model as either categorical or continuous variable separately.

### Association of peritoneal transport status and all-cause mortality by diabetic status

**Figure 2:**
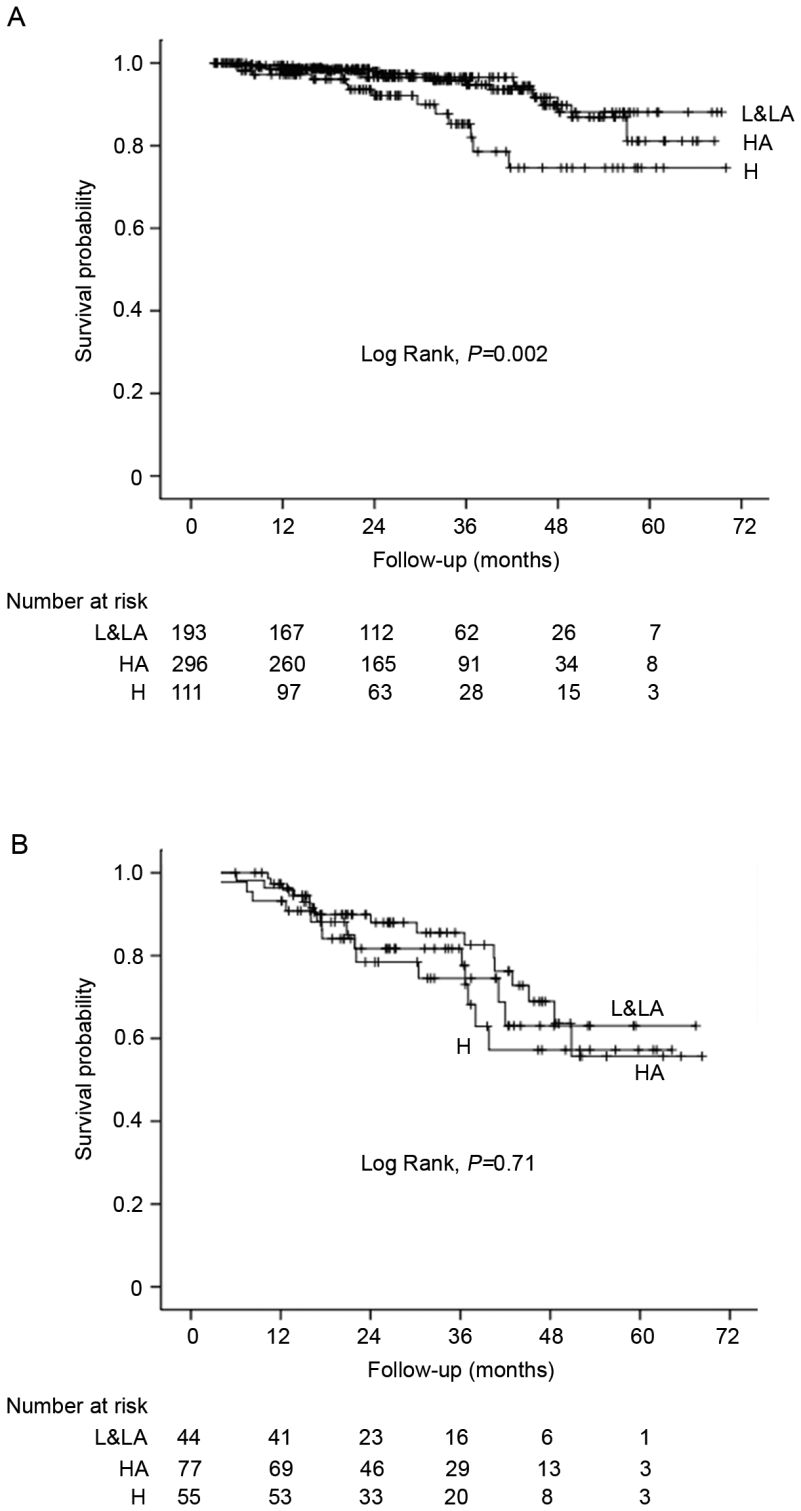
Kaplan-Meier survival curves for CAPD patients without (a) and with diabetes (b) according to PET category.

**Figure 3:**
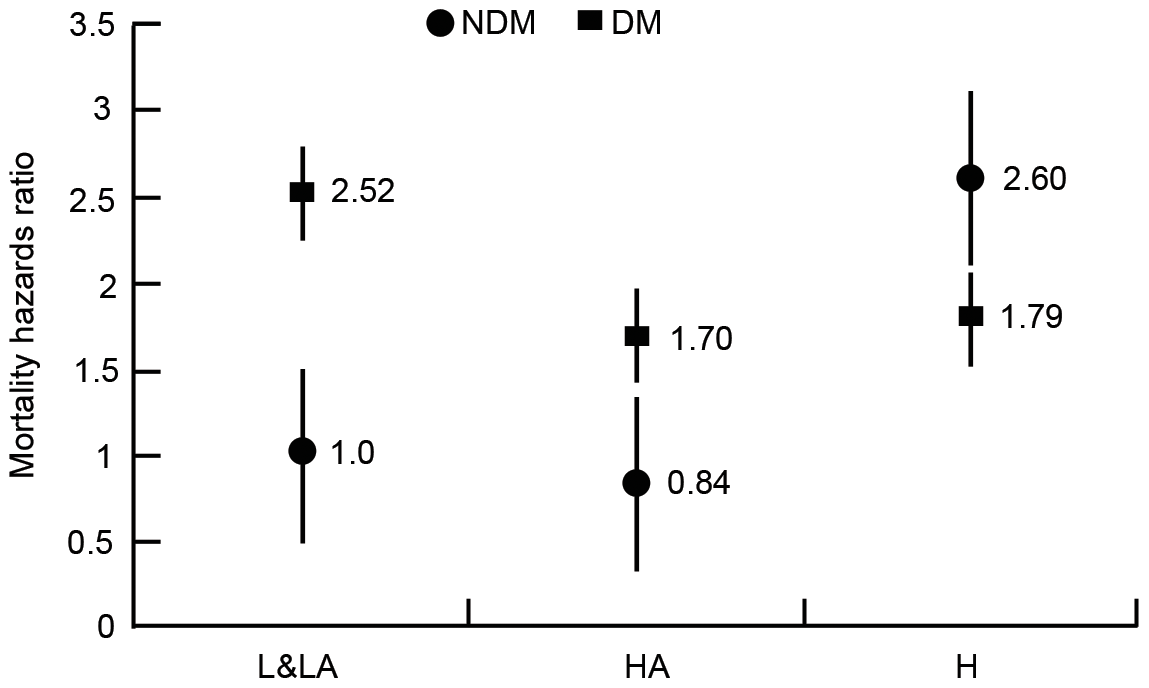
Adjusted hazard ratios of all-cause mortality for CAPD patients with and without diabetes according to PET category. Adjusted for the variables with *P*<0.1 in the univariate model, including age at initiation of peritoneal dialysis, gender, cardiovascular disease, hemoglobin, serum albumin, DBP, iPTH, total cholesterol, and triglyceride.

As shown in Figure 2, the cumulative patient survival rates among L&LA, HA and H transporters were significant different in CAPD patients without diabetes (*P* = 0.002), but not in those with diabetes (*P* = 0.71). Table 3 showed the hazard ratios of peritoneal transporters and all-cause mortality by diabetic status. In patients without diabetes, H transporters was associated with an increased risk of all-cause mortality both in unadjusted (HR 3.80, 95% CI 1.53 to 9.43; *P* = 0.004) and adjusted (HR 1.78, 95% CI 1.07 to 4.70; *P* = 0.04) analyses compared to L&LA transporters. The HR for all-cause mortality also demonstrated an enhancing trend from the L&LA to the HA to the H transporters (*P*-trend = 0.01). In contrast, mortality in DM group was not associated with transport status, no matter assessed as either categorical (adjusted HR 0.79, 95% CI 0.33 to 1.89; *P* = 0.59) or a continuous variable (D/P Cr, adjusted HR 1.11, 95% CI 0.86 to 1.42; *P* = 0.42, per 0.1 increase). Figure 3 further depicted the adjusted HRs for all-cause mortality by PET category in CAPD patients with and without diabetes. An increase trend was found between peritoneal transport status and mortality risk among patietns without diabetes, whereas no increase trend was observed in those with diabetes.

**Table 3:** Association of transport status and all-cause mortality by diabetic status.

Variable	Non-diabetic patients (n = 600)				Diabetic patients (n = 176)			
	Unadjusted model		Adjusted model*		Unadjusted model		Adjusted model*	
	HR (95% CI)	*P*	HR (95% CI)	*P*	HR (95% CI)	*P*	HR (95% CI)	*P*
Transport status as a categorical variable								
PET Class								
L&LA	Ref	–	Ref	–	Ref	–	Ref	–
HA	1.35 (0.54, 3.34)	0.51	0.75 (0.29, 1.93)	0.55	0.78 (0.36, 1.69)	0.53	0.63 (0.27, 1.42)	0.26
H	3.80 (1.53, 9.43)	**0.004**	1.78 (1.07, 4.70)	**0.04**	1.04 (0.47, 2.30)	0.92	0.79 (0.33, 1.89)	0.59
* P*- trend		**0.004**		**0.01**		0.71		0.56
Transport status as a continuous variable								
D/P Cr 4h (per 0.1 higher)	1.58 (1.18, 2.12)	**0.002**	1.49 (1.05, 2.10)	**0.03**	1.11 (0.87, 1.42)	0.40	1.11 (0.86, 1.42)	0.42

* Adjusted for the variables with *P*<0.1 in the univariate model, including age at initiation of peritoneal dialysis, gender, cardiovascular disease, hemoglobin, serum albumin, DBP, iPTH, total cholesterol, and triglyceride.

## Discussion

In the present study, we demonstrated that there was an inverse association between high peritoneal transport and all-cause mortality in CAPD population without diabetes, whereas no clear survival disadvantage was observed in those who had diabetes and in the entire cohort. These results suggested that diabetic status may modify the association of peritoneal transport with mortality. In addition, diabetes was independently associated with a higher risk of mortality in CAPD population.

Previous studies have focused on the association between peritoneal transport status and death in overall PD population, which have embraced controversially results. A study by Fried *et al* showed that the 3-yr survival probabilities gradually increased in HA, LA, and L transporters, as compared with H transporters [18]. Thereafter, CANUSA study, a prospective observation of 606 CAPD patients with 82% Caucasian in North America, demonstrated that each 0.1 increment in D/P Cr enhanced the relative risk of death by 19% [2]. Meanwhile, Davies *et al* prospectively identified that high D/P Cr was an independent predictor of mortality [5]. A meta-analysis of 20 studies also indicated there was 15% increase in the overall pooled relative risk of mortality for each 0.1 increment in D/P Cr [4]. It was difficult to apply the findings of this meta-analysis to our study, since two of the pooled studies included only continuous cycler peritoneal dialysis (CCPD) patients, seven studies targeted both CAPD and CCPD patients, and only half studies were focused on CAPD population. On the contrary, more and more investigations have failed to reach consistent results. The best known ADEMEX trial, by far the largest prospective study of 965 CAPD patients with a follow-up of 30 months, found that there was no significant effect of baseline peritoneal transport characteristics on mortality in PD patients [7]. A prospective study by Szeto *et al.* demonstrated that the low transporters had slightly better 2-year patient survival than high/high average transporters (90.2% versus 83.3%) among Chinese CAPD patients, but it was not statistically significant [19]. Similarly, the multicenter European Automated PD Outcome Study prospectively revealed that baseline D/P Cr was not associated with all-cause mortality in PD patients receiving automatic peritoneal dialysis (APD) treatment [20]. Patients using icodextrin solution also favored a significant survival advantage in high transporters [21]. Our results added the previous literature to show that there was no association between baseline peritoneal transport status and all-cause mortality in overall CAPD patients.

To our knowledge, this study is by far the first observation to date to investigate the different association of transport status and mortality between patients with and without diabetes. We found that high transporter was a risk factor for death in CAPD patients without diabetes, but not associated with an increased mortality in those with diabetes. Consistent with previous researches, high transporters showed to have a higher prevalence of hypoalbuminemia [15], [22], [24] and worse in other nutritional surrogates such as nPCR and serum prealbumin (data not shown). These suggested a greater incidence of underlying chronic inflammation and malnutrition among CAPD patients with high transporters. Nevertheless, diabetes *per se* was a significant independent risk factor for patient survival, which was in accordance with previous study [12]–[15]. Additionally, individuals with diabetes were significantly older, with greater proportion of CVD, and lower levels of serum albumin than those without diabetes, which have been reported as independently factors responsible for the poor outcomes. Thus, the aforementioned risk predictors might surpass the effects of high transporters on mortality in diabetic CAPD population.

Many studies have investigated the interaction between diabetes and inflammation, serum albumin and phosphorus, age and BMI, age and smoking status, as well as diabetes and BMI [23]–[27]. However, the interaction between transport status and diabetes on mortality risk has not been investigated in PD patients. Our data revealed a significant interaction between high transporter and diabetes, and their interactive effects attenuated the adverse impact of high transporters on patient survival among diabetic CAPD patients, suggesting that diabetes status modified the association of peritoneal transport with mortality. Plausible mechanism of the interaction was not certain. Diabetes is a disease that is strongly associated with microvascular complications, including retinopathy, nephropathy, and neuropathy [28]. The pathogenesis of microvascular disease in diabetes is multifactorial, including the production of advanced glycation end products, abnormal activation of signaling cascades, and elevated production of reactive oxygen species that may increase capillary basement membrane thickening, permeability of endothelial cells, and formation of microaneurysms. A large study by Honda et al. demonstrated that patients with diabetes showed significant increase in average postcapillary venule thickness and decrease in lumen/vessel diameter ratio compared with patients without diabetes in pre-peritoneal dialysis peritoneum [29], suggesting that diabetes-associated vascular alterations may contribute to higher proportion of high transporters in PD patients.

There are some limitations in this study. First, it was a single-center observational study, and a center-specific effect cannot be excluded. In addition, because we only enrolled CAPD patients and icodextrin was not available in the Mainland of China, thus, our results may not necessarily extend to APD patients or patients using icodextrin solution. Second, we excluded a part of patients due to unavailability of baseline PET measurements, which may carry a risk for bias. Third, relatively few patients presented low transporter status and small sample size of diabetes. Finally, our analyses did not study the relationship between the change of peritoneal transport status and mortality.

In conclusion, high peritoneal transport status confers increased all-cause mortality risk in CAPD patients without diabetes. The interaction of transport status and diabetes might modify the risk of death, and high transport did not add adverse effect on patient survival in those with diabetes. Our results should be confirmed by a large cohort of CAPD patients with diabetes and the mechanisms underlying the associations investigated.

## Supporting Information

Table S1 **Risk factors for mortality as assessed by univariate and multivariate Cox regression analysis in overall incident CAPD patients (n = 776).** *Adjusted for the variables with P<0.1 in the univariate model plus gender and RRF, including age at initiation of peritoneal dialysis, gender, cardiovascular disease, hemoglobin, serum albumin, DBP, iPTH, total cholesterol, and triglyceride and RRF. Transport status was alternatively assessed either as a categorical variable (PET classes) or as a continuous variable (D/P Cr 4h) in the multivariate adjusted model. Abbreviations: H, high transport; HA, high average transport; LA, low average transport; L, low transport; BMI, body mass index; SBP, systolic blood pressure; DBP, diastolic blood pressure; iPTH, intact parathyroid hormone; hs-CRP, high-sensitivity C-reactive protein; PET, peritoneal equilibration test; UF, ultrafitration; WCCr, total weekly creatinine clearance; RRF, residual renal function.
(DOC)
