# Supplementary material for: High Peritoneal Transport Status Was Not Associated with Mortality in Peritoneal Dialysis Patients with Diabetes
Source: PLoS One. 2014 Oct 16;9(10):e110445. doi: 10.1371/journal.pone.0110445 (PMC4199757; doi:10.1371/journal.pone.0110445)
Supplement: Table S1 — Risk factors for mortality as assessed by univariate and multivariate Cox regression analysis in overall incident CAPD patients (n = 776). *Adjusted for the variables with P<0.1 in the univariate model plus gender and RRF, including age at initiation of peritoneal dialysis, gender, cardiovascular disease, hemoglobin, serum albumin, DBP, iPTH, total cholesterol, and triglyceride and RRF. Transport status was alternatively assessed either as a categorical variable (PET classes) or as a continuous variable (D/P Cr 4h) in the multivariate adjusted model. Abbreviations: H, high transport; HA, high average transport; LA, low average transport; L, low transport; BMI, body mass index; SBP, systolic blood pressure; DBP, diastolic blood pressure; iPTH, intact parathyroid hormone; hs-CRP, high-sensitivity C-reactive protein; PET, peritoneal equilibration test; UF, ultrafitration; WCCr, total weekly creatinine clearance; RRF, residual renal function. (DOC) [file pone.0110445.s001.doc]

**Supplementary Table:**

**Table S1. Risk factors for mortality as assessed by univariate and multivariate Cox regression analysis in overall incident CAPD patients (n=776).**

| Variable | Univariate  HR (95% CI) | *P* value | Multivariate  HR (95% CI) | *P* value |
| --- | --- | --- | --- | --- |
| PET Classes |  |  |  |  |
| L&LA | Ref | **--** | Ref | **--** |
| HA | 1.07(0.60,1.92) | 0.94 | 0.74 (0.40, 1.36) | 0.33 |
| H | 2.35(1.30,4.25) | **0.01** | 1.31 (0.68, 2.54) | 0.42 |
| P trend |  | **0.003** | 0.13 |  |
| D/P Cr 4h (per 0.1 increase) | 1.42(1.17,1.72) | **0.001** | 1.18 (0.95, 1.48) | 0.13 |
| Age (per 5 years) | 1.37(126,1.50) | **<0.001** | 1.05(1.03, 1.08) | **<0.001** |
| Male gender | 1.16(0.74,1.82) | 0.34 | 0.92(0.57,1.49) | 0.74 |
| Cardiovascular disease | 2.66(1.65,4.27) | **<0.001** | 1.61(0.98,2.65) | 0.06 |
| Diabetes | 3.65(2.35,5.73) | **<0.001** | 1.62(0.98,2.71) | 0.06 |
| BMI (per 1kg/m^2^) | 1.03(1.96,1.11) | 0.45 | -- | -- |
| SBP (per 10mmHg) | 1.07(0.94,1.02) | 0.30 | -- | -- |
| DBP (per 10mmHg) | 0.72(0.60,0.88) | **0.001** | 1.03(0.82, 1.28) | 0.82 |
| Hemoglobin (per 10g/L) | 0.87(0.79,0.96) | **0.01** | 0.88(0.79, 0.99) | **0.04** |
| Serum albumin (per 10g/L) | 0.35(0.22,0.53) | **<0.001** | 0.62(0.37, 1.04) | 0.07 |
| Uric acid (per 100μmol/L) | 0.88(0.71,1.09) | 0.25 | -- | -- |
| Calcium (per 1mmol/L) | 1.10(0.78,154) | 0.59 | -- | -- |
| Phosphate (per 1mmol/L) | 0.83(0.51,1.26) | 0.34 | -- | -- |
| iPTH (per 10pg/mL) | 0.97(0.97,1.00) | **0.01** | 0.99(0.98, 1.00) | 0.08 |
| Glucose (per 1mmol/L) | 1.00(0.99,1.01) | 0.29 | -- | -- |
| Cholesterol (per 1mmol/L) | 1.02(1.01,1.04) | **0.003** | 1.04(1.00, 1.07) | **0.04** |
| Triglyceride (per 1mmol/L) | 1.12(1.05,1.18) | **0.001** | 1.13(0.96, 1.32) | 0.13 |
| hs-CRP (per 1mg/L) | 1.00(0.99,1.01) | 0.71 | -- | -- |
| UF on PET (per 100ml) | 0.91(0.81,1.03) | 0.13 | -- | -- |
| WCCr(per 1L/w/1.73 m^2^) | 1.00(0.99,1.00) | 0.81 | -- | -- |
| Weekly total Kt/V urea | 1.17(0.80,1.70) | 0.41 | -- | -- |
| RRF (per 1 ml/min/1.73 m^2^) | 0.97(0.88,1.07) | 0.58 | 0.97(0.87, 1.08) | 0.58 |

*Adjusted for the variables with P < 0.1 in the univariate model plus gender and RRF, including age at initiation of peritoneal dialysis, gender, cardiovascular disease, hemoglobin, serum albumin, DBP, iPTH, total cholesterol, and triglyceride and RRF. Transport status was alternatively assessed either as a categorical variable (PET classes) or as a continuous variable (D/P Cr 4h) in the multivariate adjusted model.

Abbreviations: H, high transport; HA, high average transport; LA, low average transport; L, low transport; BMI, body mass index; SBP, systolic blood pressure; DBP, diastolic blood pressure; iPTH, intact parathyroid hormone; hs-CRP, high-sensitivity C-reactive protein; PET, peritoneal equilibration test; UF, ultrafitration; WCCr, total weekly creatinine clearance; RRF, residual renal function.
